# Supplementary material for: The Evolution and Characterization of the RNA Interference Pathways in Lophotrochozoa
Source: Genome Biol Evol. 2024 May 7;16(5):evae098. doi: 10.1093/gbe/evae098 (PMC11114477; doi:10.1093/gbe/evae098)
Supplement: evae098_Supplementary_Data [file evae098_supplementary_data.zip › Supplementary_materials.zip!/selected_miRNAs/NoGe_selected_miRNAs/mirdeep_pdf_files/NMRB01002279.1_Notospermus_geniculatus_strain_Ushimado_scaffold2279,_whole_genome_shotgun_sequence_37114.pdf]

Provisional ID	:	NMRB01002279.1_Notospermus_geniculatus_strain_Ushimado_scaffold2279,_whole_genome_shotgun_sequence_37114
Score total	:	1221.8
Score for star read(s)	:	3.9
Score for read counts	:	1222.2
Score for mfe	:	-7.2
Score for randfold	:	0
Score for cons. seed	:	3
Total read count	:	2409
Mature read count	:	1585
Loop read count	:	0
Star read count	:	824

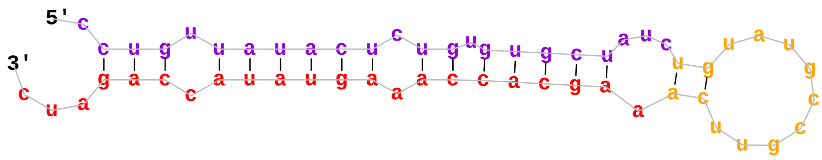

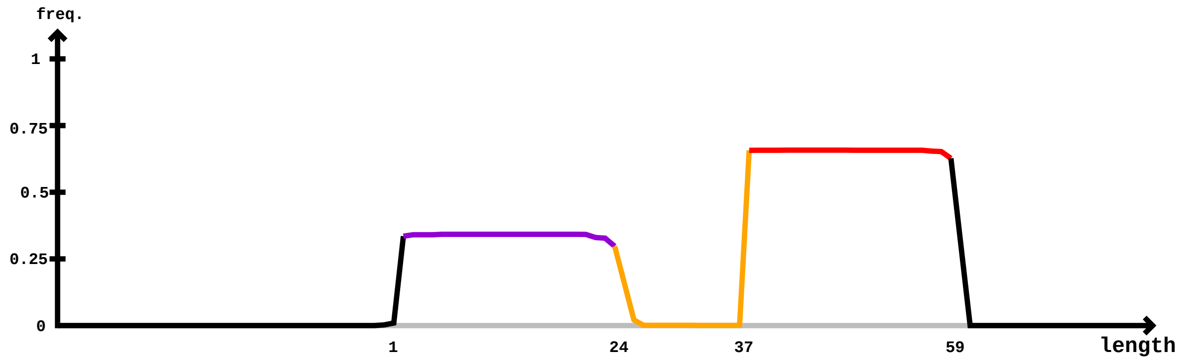

Star **Mature**

```
5'- aagaucuugcuuuuacuguccuugccacgugucuccuguuauacucugugugcuaucuguaugccguucaaagcaccaaaguauaccagaucacaaguucacugcacuugcu   -3'    obs
    aagaucuugcuuuuacuguccuugccacgugucuccuguuauacucugugugcuaucuguaugccguucaaagcaccaaaguauaccagaucacaaguucacugcacuugcu          exp
    .((((...((............)).....)))).(((.((((((.((.(((((...((.........)).))))))).)))))).)))....(((((.......)))))..    reads    mm    sample
    .................................uccuguuauacucugugugcu..........................................................    2        0     Fm3
    ..................................ccuguuauacucugugugcu..........................................................    6        0     Fm3
    ..................................ccuguuauacucugugugcua.........................................................    2        0     Fm3
    ..................................ccuguuauacucugugugcuau........................................................    6        0     Fm3
    ..................................ccuguuauacucugugugcuauU.......................................................    1        1     Fm3
    ..................................ccuguuauacucugugugcuauc.......................................................    15       0     Fm3
    ..................................ccuguuauacucugugugcuaucu......................................................    3        0     Fm3
    ......................................................................aagcaccaaaguauaccag.......................    8        0     Fm3
    ......................................................................aagcaccaaaguauaccagau.....................    16       0     Fm3
    ......................................................................aagcaccaaaguauaccagauc....................    143      0     Fm3
    ......................................................................aagcaccaaaAuauaccagauc....................    1        1     Fm3
    ......................................................................aagcaccaaaguauaccagauA....................    1        1     Fm3
    ......................................................................aagcaccaaaguauaccagauca...................    1        0     Fm3
    ......................................................................aagcaccaaaguauaccagaucU...................    3        1     Fm3

    ..................................ccuguuauacucugugugcu..........................................................    1        0     Mm3
    ..................................ccuguuauacucugugugcua.........................................................    1        0     Mm3
    ..................................ccuguuauacucugugugcuau........................................................    9        0     Mm3
    ..................................ccuguuauacucugugugcuauU.......................................................    2        1     Mm3
    ..................................ccuguuauacucugugugUuauc.......................................................    1        1     Mm3
    ..................................ccuguuauacucugugugcuauc.......................................................    41       0     Mm3
    ..................................ccuguuauacucugugGgcuauc.......................................................    1        1     Mm3
    ..................................ccuguuauacucuguguUcuauc.......................................................    1        1     Mm3
    ..................................ccuguuauacucugugugcuaucu......................................................    33       0     Mm3
    ..................................ccuguuauacucugugugcuaucG......................................................    1        1     Mm3
    ..................................ccuguuauacucugugugcuaucug.....................................................    4        0     Mm3
    ...................................cuguuauacucugugugcuaucu......................................................    1        0     Mm3
    ......................................................................aagcaccaaaguauaccagau.....................    5        0     Mm3
    ......................................................................aagcaccaCaguauaccagauc....................    1        1     Mm3
    ......................................................................Nagcaccaaaguauaccagauc....................    1        1     Mm3
    ......................................................................aagcaccaaaguauaccUgauc....................    1        1     Mm3
    ......................................................................aagcaccaaaguauacAagauc....................    1        1     Mm3
    ......................................................................aagcaccaaaguauaccagauc....................    200      0     Mm3
```

Star **Mature**

aagaucuugcuuuuacuguccuugccacgugucuccuguuauacucugugugcuaucuguaugccguucaaagcaccaaaguauaccagaucacaaguucacugcacuugcu			
....aagcaccaaaguauaccagauca...................	1	0	Mm3
....aagcaccaaaguauaccagaucU...................	5	1	Mm3
....agcaccaaaguauaccagauc....................	1	0	Mm3
....accaaaguauaccagauc....................	1	0	Mm3
.................................uccuguuauacucugugugcG..	1	1	Fm2
.................................uccuguuauacucugugugcu..	6	0	Fm2
..................................ccuguuauacucuguguUcuauc...	1	1	Fm2
..................................ccuguuauacucugugugcuauc...	44	0	Fm2
..................................ccuguuauacucugugugcuaucug...	10	0	Fm2
...................................cuguuauacucugugugcuauc...	5	0	Fm2
...................................Nuguuauacucugugugcuauc...	1	1	Fm2
....aagcaccaaaguauacUagauc....................	1	1	Fm2
....Uagcaccaaaguauaccagauc....................	1	1	Fm2
....aagcaccaaaguauaccagauc....................	142	0	Fm2
....aagcaccaaaguauacAagauc....................	2	1	Fm2
....aagcaccaaAuauaccagauc....................	1	1	Fm2
....aagcaccaaaguauaccUgauc....................	1	1	Fm2
..................................ccuguuauacucugugugcuau..	5	0	Fm1
..................................ccuguuauacucugugugcuauc...	8	0	Fm1
..................................ccuguuauacucugugugcuaucu..	3	0	Fm1
..................................ccuguuauacucugugugcuaucug...	6	0	Fm1
....aagcaccaaaguauaccagau.....................	8	0	Fm1
....aagcaccaaaguauaccagauc....................	120	0	Fm1
....aagcaccaaaguauaccUgauc....................	1	1	Fm1
....aagcaccaaaguauacAagauc....................	1	1	Fm1
....aagcaccaaaguauaccGgauc....................	1	1	Fm1
....aagcaccaaaguauaccagaucU...................	4	1	Fm1
................................cuccuguuauacucugugugcu..	1	0	Mm1
..................................ccuguuauacucuguguUcuauc...	1	1	Mm1
..................................ccuguuauacucugugugcuauc...	47	0	Mm1
..................................ccuguuauacucugAgugcuauc...	1	1	Mm1
..................................ccuguuauacGcugugugcuauc...	1	1	Mm1
..................................ccugAuauacucugugugcuaucu..	1	1	Mm1
..................................ccuguuauacucuguguCcuaucu..	1	1	Mm1
..................................ccuguuauacucugugugcuaucu..	13	0	Mm1
......................................uuauacucugugugcuaucug...	4	0	Mm1
....aagcaccaaaguauaccagau.....................	11	0	Mm1
....aagcaccaaaguauacAagauc....................	1	1	Mm1
....aagcaccaaaguauaccagauc....................	38	0	Mm1
................................cuccuguuauacucugugugc...	1	0	Mm2
................................cuccuguuauacucugugugcu..	4	0	Mm2
.................................uccuguuauacucugugugcu..	2	0	Mm2
.................................uccuguuauacucugugugcuau..	3	0	Mm2
.................................uccuguuauacucugugugcuauU...	2	1	Mm2
.................................uccuguuauacucugugugcuauc...	1	0	Mm2
..................................ccuguuauacucugugugcu..	5	0	Mm2
..................................ccuguuauacucugugugcua...	3	0	Mm2
..................................ccuguuauacucuguguUcuau..	1	1	Mm2
..................................ccuguuauacucugugugcuau..	49	0	Mm2
..................................ccuguuauacucugugugcuauU...	4	1	Mm2
..................................ccuguuauacucugugugcuauc...	237	0	Mm2
..................................ccuguuauacucugugugcuauA...	1	1	Mm2
..................................ccugAuauacucugugugcuauc...	1	1	Mm2
..................................ccuguuauacucugugAgcuauc...	1	1	Mm2
..................................ccuguuauacucuguguAcuauc...	4	1	Mm2
..................................ccuguuauacucugugGgcuauc...	1	1	Mm2
..................................ccuguuauacAcugugugcuauc...	1	1	Mm2
..................................ccuguuauacucugugugcuaucG..	2	1	Mm2
..................................ccuguuauacucugugugcuaucu..	172	0	Mm2
..................................ccuguuauacucuAugugcuaucu..	1	1	Mm2
..................................ccuguuauacucuguguUcuaucu..	1	1	Mm2
..................................ccuguuauacucugugugcuaucA..	2	1	Mm2
..................................ccuguuauacucugugCgcuaucu..	2	1	Mm2
..................................ccuguuauacucugugGgcuaucu..	1	1	Mm2
..................................ccuguuauacucuguguAcuaucu..	1	1	Mm2
..................................ccuguuauacucugugGgcuaucug...	1	1	Mm2

```
                                  Star                               Mature
aagaucuugcuuuuacuguccuugccacgugucuccuguuauacucugugugcuaucuguaugccguucaaagcaccaaaguauaccagaucacaaguucacugcacuugcu
..................................ccuguuauacucugugugcuaucug.....................................................    23    0    Mm2
..................................ccuguuauacucugugugcuaucuguaugcc...............................................    1     0    Mm2
...................................cuguuauacucugugugcuauc.......................................................    3     0    Mm2
...................................cuguuauacucugugugcuaucu......................................................    1     0    Mm2
...................................cuguuauacucugugugcuaucug.....................................................    2     0    Mm2
...........................................................uaugccguucaaagcaccaaag...............................    1     0    Mm2
.....................................................................Caagcaccaaaguauaccagauc....................    1     1    Mm2
......................................................................aagcaccaaaguauaccaga......................    4     0    Mm2
......................................................................aagcaccaaaguauaccagau.....................    21    0    Mm2
......................................................................aagcaccaaaguauaccagauc....................    796   0    Mm2
......................................................................aagcaUcaaaguauaccagauc....................    1     1    Mm2
......................................................................aagcaccaaaguauaGcagauc....................    1     1    Mm2
......................................................................aagcaccaaaguauaccagauU....................    4     1    Mm2
......................................................................aagcaccaaaguauaccagGuc....................    1     1    Mm2
......................................................................aagGaccaaaguauaccagauc....................    1     1    Mm2
......................................................................aagcaccaaaAuauaccagauc....................    1     1    Mm2
......................................................................aGgcaccaaaguauaccagauc....................    1     1    Mm2
......................................................................aagcacUaaaguauaccagauc....................    1     1    Mm2
......................................................................aagcaAcaaaguauaccagauc....................    1     1    Mm2
......................................................................aagcaccaaaguauaccUgauc....................    1     1    Mm2
......................................................................aagcaccaaaCuauaccagauc....................    1     1    Mm2
......................................................................aagcaccaaaguauaccCgauc....................    4     1    Mm2
......................................................................Uagcaccaaaguauaccagauc....................    1     1    Mm2
......................................................................aagUaccaaaguauaccagauc....................    1     1    Mm2
......................................................................aagcaccaaaguauaccGgauc....................    2     1    Mm2
......................................................................aagcaccaaaUuauaccagauc....................    2     1    Mm2
......................................................................aagcaccaaaguaCaccagauc....................    1     1    Mm2
......................................................................aagcaccaaaguaGaccagauc....................    1     1    Mm2
......................................................................aagcaccaaaguauaccagUuc....................    1     1    Mm2
......................................................................aaUcaccaaaguauaccagauc....................    1     1    Mm2
......................................................................aagcaccaaaguauacAagauc....................    2     1    Mm2
......................................................................aagcaccaaaguauaccagaucU...................    9     1    Mm2
......................................................................aagcaccaaaguauaccagauca...................    1     0    Mm2
```
